# Chimeric Porcine Deltacoronaviruses with Sparrow Coronavirus Spike Protein or the Receptor-Binding Domain Infect Pigs but Lose Virulence and Intestinal Tropism

**DOI:** 10.3390/v13010122

**Published:** 2021-01-17

**Authors:** Xiaoyu Niu, Yixuan J. Hou, Kwonil Jung, Fanzhi Kong, Linda J. Saif, Qiuhong Wang

**Affiliations:** 1 Center for Food Animal Health, Department of Animal Sciences, College of Food, Agricultural and Environmental Sciences, The Ohio State University, Wooster, OH 44691, USA; niu.214@osu.edu (X.N.); y.jacob.hou@unc.edu (Y.J.H.); jung.221@osu.edu (K.J.); fanzhikong110@hotmail.com (F.K.); saif.2@osu.edu (L.J.S.); 2 Department of Veterinary Preventive Medicine, College of Veterinary Medicine, The Ohio State University, Columbus, OH 43210, USA; 3 College of Animal Science and Veterinary Medicine, Heilongjiang Bayi Agricultural University, No. 5 Xinfeng Road, Sartu District, Daqing 163319, China

**Keywords:** porcine deltacoronavirus, S protein, reverse genetics, tissue tropism, spillover

## Abstract

Porcine deltacoronavirus (PDCoV) strain OH-FD22 infects poultry and shares high nucleotide identity with sparrow-origin deltacoronaviruses (SpDCoV) ISU73347 and HKU17 strains. We hypothesized that the spike (S) protein or receptor-binding domain (RBD) from these SpDCoVs would alter the host and tissue tropism of PDCoV. First, an infectious cDNA clone of PDCoV OH-FD22 strain (icPDCoV) was generated and used to construct chimeric icPDCoVs harboring the S protein of HKU17 (icPDCoV-S_HKU17_) or the RBD of ISU73347 (icPDCoV-RBD_ISU_). To evaluate their pathogenesis, neonatal gnotobiotic pigs were inoculated orally/oronasally with the recombinant viruses or PDCoV OH-FD22. All pigs inoculated with icPDCoV or OH-FD22 developed severe diarrhea and shed viral RNA at moderate-high levels (7.62–10.56 log_10_ copies/mL) in feces, and low-moderate levels in nasal swabs (4.92–8.48 log_10_ copies/mL). No pigs in the icPDCoV-S_HKU17_ and icPDCoV-RBD_ISU_ groups showed clinical signs. Interestingly, low-moderate levels (5.07–7.06 log_10_ copies/mL) of nasal but not fecal viral RNA shedding were detected transiently at 1–4 days post-inoculation in 40% (2/5) of icPDCoV-S_HKU17_- and 50% (1/2) of icPDCoV-RBD_ISU_-inoculated pigs. These results confirm that PDCoV infected both the upper respiratory and intestinal tracts of pigs. The chimeric viruses displayed an attenuated phenotype with the loss of tropism for the pig intestine. The SpDCoV S protein and RBD reduced viral replication in pigs, suggesting limited potential for cross-species spillover upon initial passage.

## 1. Introduction

The *Coronaviridae* family is classified into four genera: *Alphacoronavirus, Betacoronavirus, Gammacoronavirus*, and *Deltacoronavirus.* These coronaviruses (CoVs) infect various species and cause a broad spectrum of clinical outcomes, from asymptomatic infection to respiratory, gastrointestinal, hepatic, and neurological diseases, and peritonitis. Although some CoVs exhibit a narrow host spectrum, the high frequency of cross-species spillover is a well-known characteristic of the CoV family, threatening the health of both humans and domestic animals. Several studies have shown that the spike (S) gene is the main genetic determinant of CoV host, tissue, or cellular tropism [1,2,3]. Members of highly pathogenic human betacoronaviruses, including severe acute respiratory syndrome (SARS)-CoV, Middle East respiratory syndrome (MERS)-CoV and the causative agent of the COVID-19 pandemic, SARS-CoV-2, originated from bats and were transmitted to humans directly or indirectly through one or several intermediate hosts [4,5,6,7,8,9,10]. 

Porcine deltacoronavirus (PDCoV) was initially found in pig fecal samples collected in Hong Kong in 2009 and confirmed as the etiologic agent causing diarrhea in pigs in the United States in 2014 [11,12]. Although when and how PDCoV emerged in the swine population remains unclear, it is proposed that birds have served as the major reservoir for the gene source and fueled the evolution and dissemination of deltacoronaviruses (DCoVs) [11,13]. It is known that wild birds may serve as critical spreaders in influenza virus transmission [14,15,16,17]. Upon certain conditions, including environmental and physiologic stress, bird migration has shown a strong association with influenza outbreaks [14]. For DCoVs, one SpDCoV strain, HKU17, was sequenced and showed over 90% amino acid identity to PDCoV for seven conserved replicase domains, suggesting that SpDCoV HKU17 and PDCoV are subspecies of the same species [11]. Furthermore, SpDCoV strains were detected from sparrow fecal samples collected from swine farms in the United States [18] and they showed higher sequence identity to PDCoV and HKU17 than to other avian DCoVs, suggesting that there may be an evolutionary association between the SpDCoVs and PDCoV. Also, various avian and non-swine cell lines are susceptible to PDCoV infection [19,20]. Thus, PDCoV exhibits a broad cell tropism, infecting various cell lines derived from multiple species, including humans, pigs, chickens, and cattle. In addition, PDCoV also utilizes a conserved region of host aminopeptidase N (APN) of different species for viral attachment and entry process [19,21,22]. Besides the cellular tropism, PDCoV experimentally infected poultry [13] and gnotobiotic calves [23]. Collectively, both in vitro and in vivo studies suggeste a broad host range of PDCoV.

DCoVs has the smallest RNA genome among all known CoVs (26 kb) with a similar genomic organization to that of other CoVs: ORF1ab, S (spike) protein, E (envelop) protein, M (membrane) protein, N (nucleocapsid) protein, and accessory proteins [11,18,24]. The S protein facilitates binding to the host receptor and membrane fusion in the CoV entry process, making it the primary determinant in CoV tropism. The S protein of a CoV can be divided into two functionally distinct subunits: S1 subunit and S2 subunit [25,26]. The C-terminal domain (CTD) of the S1 subunit of PDCoV S protein was identified as the receptor-binding domain for APN [27]. Cryo-electron microscopy suggested that PDCoV-CTD showed identical structural folds to those of alphacoronavirus [28]. The alphacoronavirus transmissible gastroenteritis coronavirus (TGEV) causes severe diarrhea in young pigs. A naturally occurring variant of TGEV, the porcine respiratory coronavirus (PRCV), showed changes in tissue tropism with a large deletion and point mutations in S protein and alteration in the ORF3 [29,30]. PRCV is non-enteropathogenic but causes respiratory infection and disease in pigs [31]. The TGEV/PRCV scenario suggests that changes within the PDCoV S protein may lead to altered host or tissue tropism.

As the viral membrane protein responsible for cell entry, CoV S proteins bind to receptors on target cells and mediate subsequent virus-cell fusion [32]. Therefore, highly variable regions identified in the S protein may be responsible for the distinct host range for PDCoV and SpDCoV. We hypothesized that the recombinant PDCoVs with S protein or the RBD of the S protein replaced with that of SpDCoVs can lead to altered tropism. To test that hypothesis, an infectious cDNA clone of PDCoV was constructed. Next, recombinant PDCoVs with engineered S proteins were generated and used to inoculate neonatal gnotobiotic (Gn) pigs to evaluate their pathogenesis.

## 2. Materials and Methods

### 2.1. Cell Lines and Viruses

LLC porcine kidney (LLC-PK1) cell (ATCC CL-101) were cultured in modified Eagle’s medium (MEM, Life Technologies, Carlsbad, CA, USA) supplemented with 5% fetal bovine serum (FBS, Hyclone, Logan, UT, USA), 1% nonessential amino acids (NEAA, Life Technologies, Carlsbad, CA, USA), 1% antibiotic-antimycotic (Life Technologies, Carlsbad, CA, USA), and 1% HEPES (Life Technologies, Carlsbad, CA, USA). After PDCoV inoculation and adsorption for one hour at 37 °C with 5% CO_2_, the LLC-PK1 cells were maintained in MEM supplemented with 1% antibiotic-antimycotic, 1% NEAA, 1% HEPES, and 10 μg/mL trypsin (Life Technologies, Carlsbad, CA, USA) [33]. Swine testis (ST, ATCC CRL1746) and ST-APN-knockout (KO) cells [19], kindly provided by Dr. Berend Jan Bosch at Utrecht University, were maintained in Dulbecco modified Eagle medium (DMEM, Life Technologies, Carlsbad, CA, USA) supplemented with 10% FBS. PDCoV virulent strain OH-FD22 at cell culture passage level 7 (P7) was used as the virulent control in the Gn pig study and its genome sequence was used as the backbone for the infectious cDNA clone construction [12].

### 2.2. Structural Modeling 

The 3D structure of S proteins of recombinant viruses and SpDCoV ISU73347 were modeled with SWISS-MODEL (https://swissmodel.expasy.org) using PDCoV S protein (SMTL ID: 6bfu.1) as the template. The structural analysis was carried out with UCSF Chimera (http://www.rbvi.ucsf.edu/chimera). 

### 2.3. Construction of the Full-Length cDNA Clone of OH-FD22 and the Recovery of Recombinant Viruses

**Figure 1:**
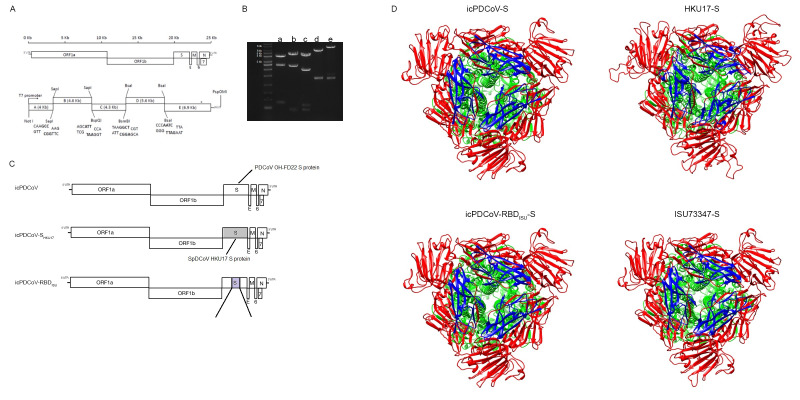
Schematic of the full-length porcine deltacoronavirus (PDCoV) genome and construction of the infectious PDCoV cDNA clone. (**A**) The upper panel is the PDCoV genome, including ORF1a, ORF1b, and the spike (S), envelope (E), membrane (M), ORF6, ORF7, and nucleocapsid (N) genes. The lower panel is cDNA fragments comprising icPDCoV. Restriction sites joining fragments are shown. (**B**) Lanes a, b, c, d, and e correspond to the agarose electrophoresis of the digested plasmids containing fragments A, B, C, D, and E. (**C**) Diagram of engineered S protein in chimeric viruses. (**D**) 3D model of the structure of S proteins homo-trimer from recombinant viruses and SpDCoV ISU73347. [S1 domain (in red), S2 domain (in green), RBD (in blue)].

The infectious cDNA clone of PDCoV OH-FD22 was constructed using similar methods to a porcine epidemic diarrhea virus (PEDV) reverse genetics platform described previously [34]. The viral RNA of the tissue culture-adapted OH-FD22 strain P7 was extracted using the RNeasy Mini kit (Qiagen, Hilden, Germany) and reverse transcribed into cDNA using SuperScript III reverse transcriptase (Invitrogen, Carlsbad, CA, USA). The genomic cDNA was divided into five fragments (Figure 1). The five fragments A to E were amplified by PCR with PrimeSTAR GXL polymerase (TaKaRa, Kyoto, Japan) and cloned into high-copy-number plasmid pUC19 (NEB, Ipswich, MA, USA). The junction between A/B fragments was joined by a unique nonpalindromic SapI site located at nucleotide positions 3988. The B/C and C/D junctions were divided by the cleavage sites located at nucleotide position 8622 and 12903, respectively. Fragments D and E were divided by a BsaI site located at nucleotide position 18468. A naturally occurring BsaI site, located within N gene, was removed by introducing synonymous mutations (G24597C). Fragment A contained a T7 promoter located upstream of the first nucleotide, whereas fragment E harbored a 25-nt poly(A) tail at the 3′ end.

Each plasmid was transformed into chemically competent *E. coli*. cells and extracted when it grew to a high concentration. After that, the harvested plasmids were subjected to enzyme digestion. The appropriately sized cDNA inserts were purified using the QIAquick gel extraction kit (Qiagen, Hilden, Germany). All five fragments were ligated with T4 ligase (NEB, Ipswich, MA, USA) at 4 °C overnight in an equal molar ratio. The ligation products were purified by chloroform extraction and precipitated using ethanol. After that, the purified full-length cDNA was subjected to in vitro transcription using mMessage mMachine T7 transcription kit (Ambion, Austin, CA, USA). The polyadenylated PDCoV N gene transcript was generated with an inserted T7 promoter and co-electroporated into the cells with the full-length transcripts at 450 V and 50 μF using a Gene Pulser II electroporator (Bio-Rad, Hercules, CA, USA). After recovery in growth medium with FBS for 24 h, the cells were washed with (phosphate-buffered saline (PBS) twice and cultured in the maintenance medium in the presence of 10 µg/mL trypsin for 3~5 days. When 80% of cells showed cytopathic effects, the supernatant was collected and designed as icPDCoV at passage level 0 (P0); this P0 virus was used for plaque purification.

The SpDCoV strains HKU17 (GenBank: NC_016992.1) and ISU73347 (GenBank: MG812378.1) were used as the reference sequences where the engineered S protein or the RBD were synthesized from Bio Basic Inc. (Amherst, NY, USA). PDCoV S gene within the plasmid E was replaced by that of SpCoV HKU17; the construction was designated as E-S_HKU17_. Moreover, the RBD of PDCoV S gene was replaced with the counterpart of SpCoV ISU73347, generating the plasmid E-RBD_ISU_. Each E fragment, either containing wild-type S or mutated S gene, together with the fragments A to D was used to generate the chimeric viruses icPDCoV, icPDCoV- S_HKU17_, and icPDCoV-RBD_ISU_.

### 2.4. Plaque Assay

LLC-PK1 cell monolayers in six-well plates were washed with maintenance medium and inoculated with the 10-fold serially diluted virus for one hour at 37 °C. After that, the inoculum was removed, and cells were washed with PBS. Then cell monolayers were covered with 2 mL/well of overlay containing 1.5% agarose as described previously [35]. Plates were incubated for 96 h. At 96 hpi, the overlay was removed, and cells were fixed with 10% PBS-buffered formalin and stained with 0.2% crystal violet.

### 2.5. Viral Growth Kinetics

LLC-PK1 cell monolayers in six-well plates were infected with the corresponding viruses at a multiplicity of infection (MOI) of 0.01. After a one-hour absorption, the cell monolayers were washed twice with PBS and then cultured in the maintenance medium as described above. The supernatants were collected at multiple time points, and the virus titers were determined in 96-well plates as 50% tissue culture infectious doses (TCID_50_) by the Reed-Muench method [36].

### 2.6. Infection of the Recombinant Viruses in Wildtype ST (ST-WT) Cells and ST-APN-KO Cells

ST-WT or ST-APN-KO cell monolayers were washed twice with PBS, followed by inoculation with the different viruses with an MOI = 0.2 for one hour. TGEV (Miller strain) and PEDV (PC22A strain), originated from our labs, were used as controls [37,38]. After removal of the inoculum, the cells were washed twice with PBS and then maintained in fresh DMEM supplemented with 0.5 μg/mL trypsin. Cells were cultured for an additional 8h and subjected to immunofluorescent (IF) for the detection of PEDV, PDCoV N proteins, or TGEV S protein.

### 2.7. Study Design of the Experimental Infection of Gn Pigs

To evaluate the pathogenesis of the recombinant viruses, experimental inoculation was performed in Gn pigs as described previously [37]. All experiments carried out in this study were approved by the Institutional Animal Care and Use Committee (IACUC) of The Ohio State University. Seventeen Gn pigs were surgically derived from two PDCoV-free sows and randomly divided into 6 groups. Piglets in the same groups were housed in the same 1–2 isolators. At 4 days of age, the piglets were inoculated with icPDCoV (orally, *n*  =  4; 10^8^ PFU/pig), icPDCoV-S_HKU17_ [orally, *n*  =  3; 10^8^ PFU/pig or oronasally (inoculum ratios: oral route/nasal route = 2/1), *n* = 2; 10^8^ PFU/pig], icPDCoV-RBD_ISU_ (orally, *n*  =  2; 10^8^ PFU/pig), OH-FD22 as virulent PDCoV control (orally, *n*  = 3, 10^8^ PFU/pig), or MEM as mock (orally, *n* = 2). One pig, as a commingled contact, was kept in the same isolator with two icPDCoV-S_HKU17_ inoculated pigs. Clinical signs, including diarrhea and vomiting, were monitored daily. Rectal swabs were collected daily to determine the severity of diarrhea and for fecal viral shedding detection. The severity of diarrhea was scored based on the fecal consistency in individual pigs: 0, solid; 1, pasty; 2, semiliquid (mild diarrhea); and 3, liquid (severe diarrhea). Nasal swabs were collected between 1–4 days post-inoculation (dpi). At the acute phase of infection, one piglet from the OH-FD22 group and two from the icPDCoV group had severe diarrhea and high viral RNA shedding. These three pigs were euthanized for histopathological examination. The remaining pigs were euthanized at 11 dpi when no viral RNA shedding was detected for at least three consecutive days.

### 2.8. RNA Extraction, Sequencing, and TaqMan Real-Time Reverse-Transcription Quantitative PCR (RT-qPCR)

Rectal and nasal swab samples were collected and subjected to total RNA extraction using MagMax RNA isolation kit (Austin, CA, USA) and MagMAX™ Express instrument (Thermofisher, Waltham, MA, USA) according to the manufacturer’s instructions. After that, the PDCoV RNA titers of swab samples were determined by RT-qPCR using the OneStep RT-PCR Kit (QIAGEN, Valencia, CA, USA) targeting the PDCoV M gene with primers (forward 5′-ATCGACCACATGGCTCCAA and reverse 5′-CAGCTCTTGCCCATGTAGCTT) and the probe (FAM-5′-CACACCAGTCGTTAAGCATGGCAAGCT-IABkFQ) as described previously [39]. To confirm the viral shedding from the respiratory tract of pigs inoculated with recombinant viruses, the RNAs of nasal swab samples showed positive in RT-qPCR were reverse-transcribed using SuperScript^®^ IV Reverse Transcriptase (Thermofisher, Waltham, MA, USA) and subjected to sanger sequencing.

### 2.9. Immunohistochemistry (IHC) and Immunofluorescent IF Staining

At necropsy, duodenum, jejunum, ileum, colon, and other major organs (heart, liver, turbinate, lungs, kidneys, spleen, and mesenteric lymph nodes) were collected and fixed in 10% PBS-buffered formalin. All tissues, except turbinate, were trimmed, processed, embedded in paraffin, and sectioned with routine procedures [40]. Because of the hardness of turbinate, it was subjected to de-calcification in 10% buffered EDTA (pH = 7.4) for one week. When it became soft enough for sectioning, turbinate samples were fixed in 10% formalin and processed as previously described for IHC staining [41]. Tissues were counterstained with hematoxylin. Mouse monoclonal antibody (mAb) SD55-197 against PDCoV N proteins (www.medgenelabs.com) was used as the primary antibody for the detection of PDCoV-infected cells. The IHC staining procedure was performed as described previously, using a non-biotin polymerized horseradish peroxidase system (BioGenex Laboratories, San Ramon, CA, USA). Ratios of villous height to crypt depth (VH/CD) of the jejunum and ileum of each group were measured as described before [40]. For each intestinal section, 8 villi and crypts were selected and measured from different sections.

For IF staining, cells were fixed and permeabilized with 100% ice-cold methanol at 24 hpi. Then cells were stained with primary antibodies (SD55-197 for PDCoV N protein and SD6-29 for PEDV N protein, kindly provided by Dr. Eric Nelson, South Dakota State University, and 25C9.3 for TGEV S protein) at 4 °C overnight. After washing with PBS for three times, Alexa Fluor 488 (AF488)-conjugated goat anti-mouse IgG (Invitrogen, Carlsbad, CA, USA) was added as the secondary antibody. Cells were incubated for 1 h at room temperature. After washing with PBS, 1 mg/mL of DAPI (4′,6-Diamidino-2-Phenylindole, Dihydrochloride, Roche Life Science, Indianapolis, IN, USA) was added for nucleic acid staining and images of stained tissues were captured by Olympus IX-70 fluorescent microscope (Olympus, Tokyo, Japan).

### 2.10. Statistical Analysis

The statistical analyses were performed using GraphPad Prism, version 8. Comparisons of diarrhea rates were analyzed by one-way analysis of variance (ANOVA). A *p*-value of less than 0.05 was considered significantly different. The onset of diarrhea, mean cumulative fecal consistency score of each pig, duration of diarrhea, peak nasal RNA shedding titers, the onset of fecal RNA shedding, and mean peak fecal RNA shedding titers were shown in Mean ± Standard Deviation (SD).

## 3. Results

### 3.1. Recombinant Viruses Were Rescued and Characterized in Cell Culture

**Figure 2:**
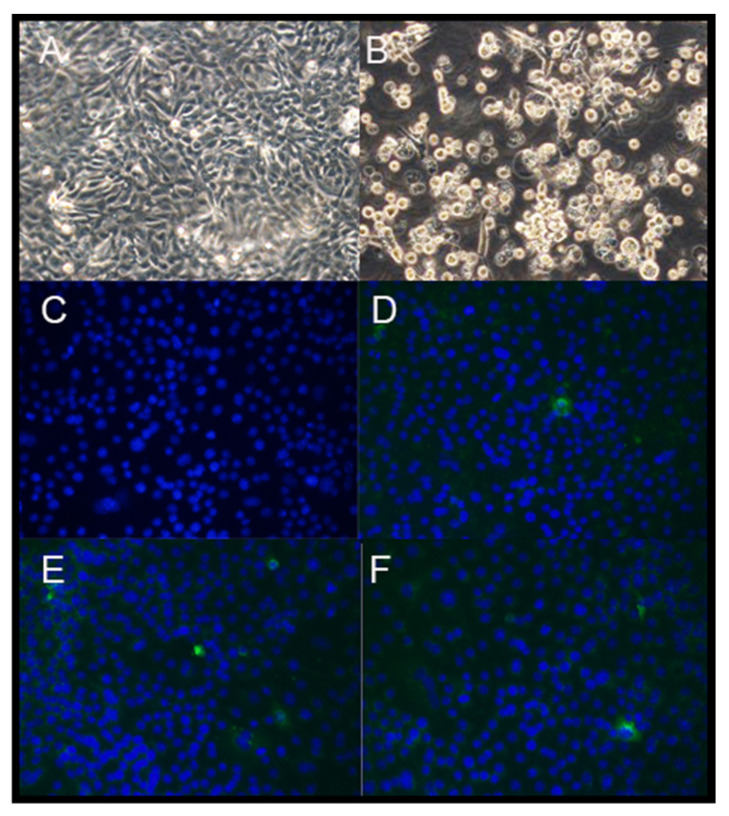
The rescue of the recombinant PDCoVs (magnification, 100×). LLC-PK1 cells transfected with N transcript only (**A**) and the full-length mRNA genome and N transcript of PDCoV (**B**) at 72 h post electroporation (hpi) viewed by light microscopy. IF staining for cells inoculated with control (**C**), icPDCoV (**D**), icPDCoV-RBD_ISU_ (**E**), and icPDCoV-S_HKU17_ (**F**) at 24 hpi [PDCoV N gene (in green) and nuclei (in blue)].

**Figure 3:**
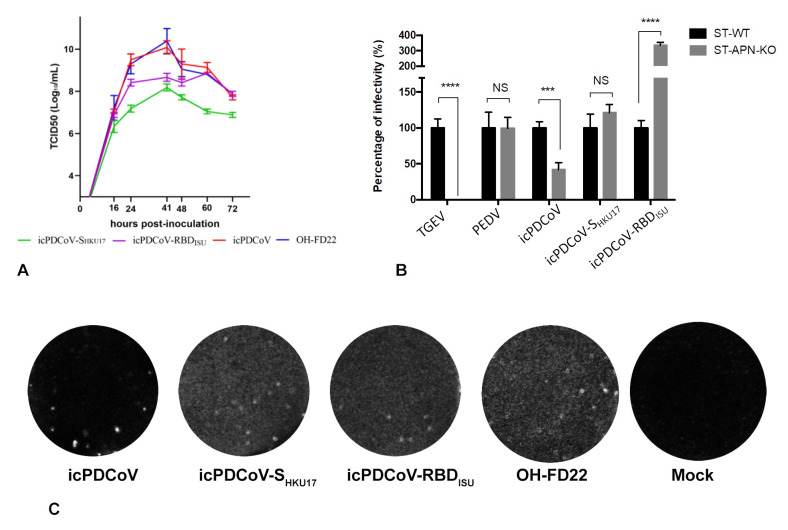
Characterization of the recombinant PDCoVs. (**A**) Multistep growth kinetics of the infectious clone-derived recombinant PDCoVs (icPDCoV, icPDCoV-RBD_ISU_, icPDCoV-S_HKU17_), and the parental PDCoV OH-FD22 strain. LLC-PK1 cells were inoculated with each virus at a MOI of 0.01. Supernatants were sampled at different time points and titrated for TCID_50._ (**B**) The infectivity of the recombinant viruses in wildtype ST (ST-WT) cells and APN-knockout ST (ST-APN-KO) cells. TGEV and PEDV were used as APN-dependent and APN-independent controls, respectively. Monolayer of cells infected with different viruses with MOI = 0.2. After removal of the inoculum, the cells were cultured for additional 8 h, fixed and stained for TGEV, PEDV, and PDCoV, respectively. Fluorescent focus units (FFU) were counted using a fluorescent microscope. Data are presented as the means ± SD. *** *p* < 0.005; **** *p* < 0.001; NS, no significance. (**C**) Plaques of recombinant PDCoVs in LLC-PK1 cells. Cells were fixed and stained at 96 hpi.

The transcripts of the PDCoV full-length genome and N gene were mixed and co-electroporated into LLC-PK1 cells. Obvious syncytia, characterized as the typical cytopathic effect (CPE) of coronaviruses, were observed at 72 h after electroporation. All supernatants were collected when 80% of the cells showed CPE and designated as P0. The P0 viruses were plaque purified and sequencing confirmed as designed. The infectious titers of the P1 viruses were 7.63 ± 0.94, 8.07 ± 0.40, and 10.29 ± 0.54 log_10_ TCID_50_/mL for icPDCoV-S_HKU17_, icPDCoV-RBD_ISU_, and icPDCoV, respectively. Moreover, the virus replication was confirmed by IF staining of PDCoV N proteins in icPDCoV-P1-infected cells (Figure 2). The replication of recombinant viruses was assessed by multistep growth kinetics at a MOI of 0.01. Though all three recombinant viruses replicated at a similar rate at the early phase [within 16 h post-inoculation (hpi)], much lower peak infectious titers were observed for icPDCoV-S_HKU17_ and icPDCoV-RBD_ISU_ mutants (Figure 3A). In summary, those two chimeric viruses showed less efficient replication compared with icPDCoV in LLC-PK1 cells.

### 3.2. Recombinant icPDCoVs with Distinct S Counterparts Differed in Their Replication in APN-KO Cells

Coronavirus S protein, responsible for the receptor-binding function, initiates the viral infection cycle and determines the host range and tissue tropism. To investigate whether altered S protein leads to any variation in receptor usage, we infected either ST cells or ST-APN-KO cells with icPDCoV and chimeric viruses. TGEV and PEDV were used as controls as infection is dependent and independent of APN, respectively. Unlike TGEV, whose infection was completely abolished, icPDCoV replication partially decreased due to the loss of APN in ST cells (Figure 3B), consistent with a previous report [19]. However, the presence of APN did not significantly affect icPDCoV-S_HKU17_ infection of ST cells, like the PEDV control. For icPDCoV-RBD_ISU_, increased infection in ST cells was observed upon the knockout of APN. The results suggest that S counterparts from the chimeric viruses showed different preferences of viral entry strategies and PDCoV may also use other host factors as the receptors.

### 3.3. icPDCoV Showed Comparable Pathogenesis in Neonatal Gn Pigs as OH-FD22 and Chimeric Mutants Exhibited Nasal but Not Fecal Viral RNA Shedding

**Figure 4:**
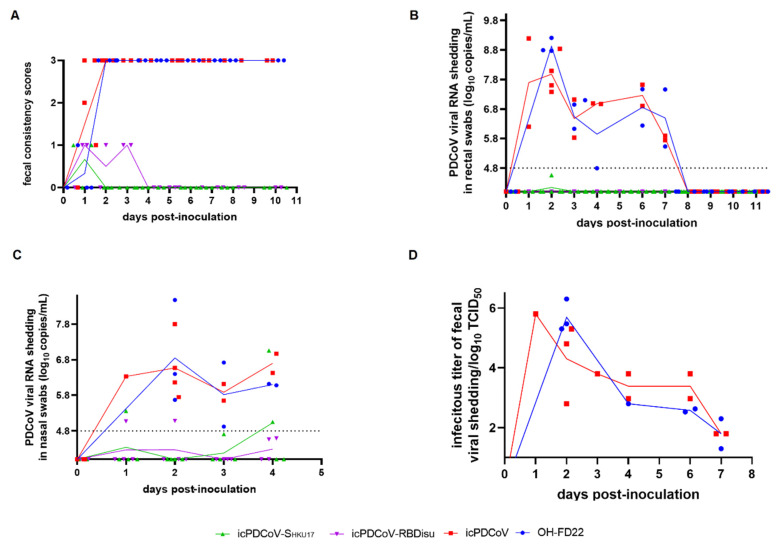
Evaluation of the replication and pathogenesis of recombinant viruses icPDCoV, icPDCoV-S_HKU17_ and icPDCoV-RBD_ISU_ in 4-day-old Gn piglets. OH-FD22 group was positive control. Each line represents the mean value of each group and the values of individual Gn piglets are also shown: (**A**) Fecal consistency scores (0, solid; 1, pasty; 2, semiliquid; and 3, liquid. score of ≥2 was considered diarrhea.), (**B**) Fecal PDCoV RNA shedding profile, (**C**) Nasal PDCoV RNA shedding profile, and (**D**) Infectious PDCoV shedding titers from fecal samples.

We investigated the pathogenesis of the recombinant PDCoVs in neonatal Gn pigs (Table 1). After virus inoculation, all pigs in OH-FD22- and icPDCoV-inoculated groups had severe diarrhea by 2 dpi, with similar peak fecal viral RNA levels (8.92 ± 0.25 log_10_ copies/mL for OH-FD22 and 8.38 ± 0.81 log_10_ copies/mL for icPDCoV) or infectious virus shedding titers (5.69 ± 0.54 log_10_ TCID_50_/mL for OH-FD22 and 4.63 ± 0.76 log_10_ TCID_50_/mL for icPDCoV) (Figure 4B,D). However, pigs in the chimeric virus groups showed no diarrhea or other clinical signs. No fecal viral RNA shedding was detected in the icPDCoV-S_HKU17_ or icPDCoV-RBD_ISU_ groups until 11 dpi (Figure 4B). Furthermore, no clinical signs or viral shedding were evident for the pig commingled with the icPDCoV-S_HKU17_ group. In addition to the fecal viral RNA shedding, all OH-FD22- and icPDCoV-inoculated pigs showed low-moderate levels of viral RNA shedding in nasal swabs (4.92–8.48 log_10_ copies/mL and 5.65–7.80 log_10_ copies/mL, respectively) (Figure 4C). However, nasal viral RNA shedding was detected in 40% (2/5) of pigs in icPDCoV-S_HKU17_ group (5.36–7.06 log_10_ copies/mL) and 50% (1/2) of pigs in icPDCoV-RBD_ISU_ group (5.07–5.08 log_10_ copies/mL). The nasal viral shedding in the chimeric virus groups showed an increasing trend from 1–4 dpi. Moreover, Sanger sequencing showed that the viral RNA from nasal swabs of the chimeric virus-inoculated pigs shared identical S genes with the original chimera inoculum. These results suggested limited replication of chimeric viruses in the pig respiratory tract.

**Figure 5:**
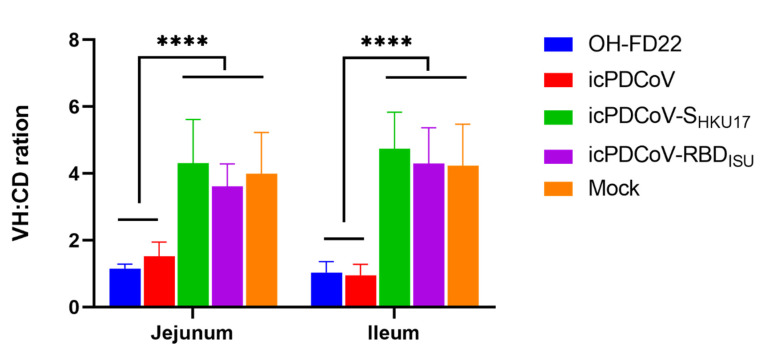
Villous height to crypt depth (VH:CD) ratios of piglets inoculated with recombinant viruses icPDCoV, icPDCoV-S_HKU17_ or icPDCoV-RBD_ISU_. OH-FD22 and mock groups were controls. Groups with significant differences (**** *p* < 0.0001) are indicated with different letters.

**Figure 6:**
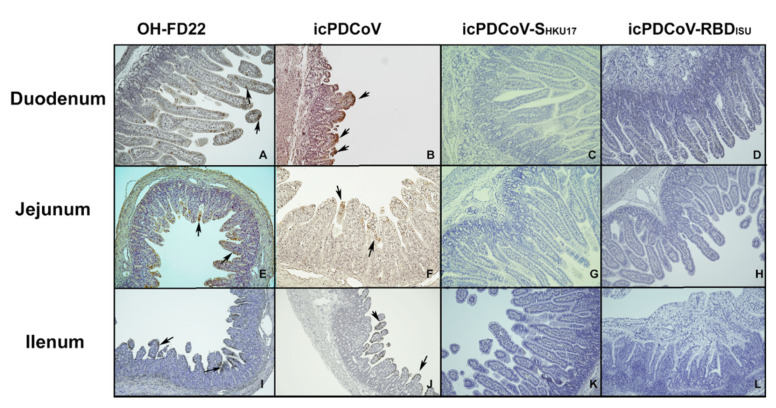
Immunohistochemistry staining of PDCoV N proteins in the enterocytes of duodenum, jejunum, and ileum of piglets inoculated with OH-FD22 (**A**,**E**,**I**), icPDCoV (**B**,**F**,**J**), icPDCoV-S_HKU17_ (**C**,**G**,**K**), or icPDCoV-RBD_ISU_ (**D**,**H**,**L**) (magnification, 100×). The brown signals, indicated by arrows, represent the PDCoV antigens in enterocytes and were observed in the OH-FD22- and icPDCoV-inoculated, but not in icPDCoV-S_HKU17_- or icPDCoV-RBD_ISU_-inoculated and mock piglets (data not shown).

Two pigs from the icPDCoV group and one from the OH-FD22 group showing severe clinical signs and two pigs in mock group were euthanized at 3 dpi. At necropsy, severe atrophic enteritis was observed in the virus-inoculated pigs. The intestinal walls of different sections of small intestines were thin and transparent. Some yellow fluid was accumulated in the intestinal lumen. IHC staining showed extensive PDCoV N proteins in the remaining epithelial cells of the small intestine, especially in ileum and jejunum. VH: CD ratio of villi was used to describe the severity of villous atrophy of the infected pigs. Inoculation of OH-FD22 or icPDCoV induced comparable severe villous atrophy (Figure 5). However, no significant villous atrophy was observed in the pigs inoculated with the two chimeric viruses (Figure 5). In the chimeric virus groups, no lesions were observed in the intestinal tract, and no PDCoV N protein was detected by IHC staining of the intestinal sections (Figure 6C,D,H,I,M,N). These data indicate that icPDCoV virus is highly pathogenic in newborn Gn piglets, like virulent PDCoV strain OH-FD22, and the two chimeric viruses lost intestinal tropism, replicated poorly in nasal cavity, and did not cause disease in pigs.

## 4. Discussion

PDCoV was first identified in swine in a surveillance study in 2012 [11], but its etiological role in swine disease was not determined until 2014 [42]. In Feb 2014, PDCoV infection was detected in five Ohio swine farms experiencing an outbreak of PEDV-like diarrhea disease [42]. Once PDCoV was identified as the pathogenic cause of diarrheal diseases in pigs in the United States, Wang et al. reported PDCoV positive pigs in nine of 10 major pig-producing US states, from which feces and intestine samples were collected [43]. Even though PDCoV does not cause high mortality in piglets as does PEDV, sick pigs carrying virus may serve as spreaders potentially causing much more extensive transmission. As a potential emerging zoonotic pathogen [19], it is urgent to provide an advanced platform for PDCoV research and vaccine development. The infectious full-length cDNA based molecular clone for a US virulent PDCoV strain, OH-FD22, was constructed in this study. Both the virulent parental PDCoV OH-FD22 strain and the infectious clone-derived virus were highly pathogenic in neonatal Gn pigs. Thus, this infectious PDCoV clone provides a platform for the assessment of viral gene function and the development of live attenuated vaccines (LAVs). Upon the development of the reverse genetics platform, we plan to expand research on this unique pathogen to (1) engineer mutants to examine viral gene functions in vitro and in vivo, (2) develop recombinant viruses to investigate the mechanisms of broad host ranges of DCoVs, and (3) develop LAV candidates against PDCoV. 

Although it is still unknown whether PDCoV was introduced into North America following its emergence and circulation in Asia, where no association of infection with clinical disease was reported [44], the decreased replication and pathogenicity in pigs observed in Asia suggests that PDCoV has spilled over to swine recently from birds and is incompletely adapted to pigs. In this study, chimeric PDCoVs carrying the S protein of HKU17 or the RBD of ISU73347 SpDCoV strains, which are genetically closest to PDCoVs, were generated to investigate whether such viruses can infect pigs. However, the chimeric viruses with the intact S protein from ISU73347 were nonviable. Consequently, the RBD from ISU73347 was used to design the chimeric virus and a viable icPDCoV-RBD_ISU_ was generated. This data suggests that HKU17 may be more closely related to PDCoV than ISU73347 and the S gene from ISU73347 may play an ancestral role, compared with HKU17, for DCoV emergence in pigs. The experimental infection indicated that chimeric viruses bearing the S gene or RBD from SpDCoVs do not replicate in the enteric tract of pigs as PDCoV does, but interestingly the ability to replicate in the respiratory tract is retained. Variations in CoV S gene leading to changes in host or tissue tropism have been well characterized in other genera of CoVs. TGEV replicates more efficiently in the villus epithelial cells of small intestine and less in the epithelial cells of the upper respiratory tract; it causes severe enteric disease but not obvious respiratory symptoms as the first swine enteropathogenic CoV [45]. PRCV, a naturally occurred genetic variant of TGEV, was identified in 1984 [46]. However, PRCV is a non-enteropathogenic virus and exhibits robust replication in the respiratory tract. Ballesteros et al. suggested that two nucleotide changes at positions 214 and 655 of the S gene might be responsible for the loss of the enteric tropism of the PTV-ts-mad TGEV strain [47]. However, the APN binding region of the RBD of PRCV is retained and is identical to TGEV. Data from others indicated that loss of enteric tropism of PRCV was due to loss of the sialic acid binding domain that is needed to retain intestinal tropism and gut infection [48,49]. In our study, pigs inoculated with chimeric icPDCoVs did not have diarrhea, detectable fecal viral shedding, or viral N protein in intestinal epithelial cells, suggesting that chimeric viruses were non-enteropathogenic and the replaced S protein or RBD from SpDCoV was responsible for the abolished tropism in the enteric tract. In addition to tissue tropism changes caused by S protein mutation, various CoVs have spilled over into a novel host upon S protein changes. For example, SARS-CoV, MERS-CoV, and SARS-CoV-2 are zoonoses, in which bats serve as the original CoV gene source [50,51,52]; the intermediate hosts of which were civet cats [53], camels [54], and unknown animals [55], respectively. Molecular clock analysis suggested that birds may be ideal hosts for the DCoV gene source, which contributed to DCoV evolution, dissemination, and potential spillovers. Although we did not observe robust viral replication in pigs inoculated with the chimeric viruses with SpDCoV-origin S protein/RBD counterparts, those recombinant viruses did replicate well in vitro on the pig-origin cell line LLC-PK1. The reason for these differences may be that the SpDCoVs HKU17 and ISU73347 strains used as gene sources in this study were not the direct donors for the S and RBD gene of PDCoV. 

In our study, either an intact PDCoV S gene or the RBD was replaced by the SpDCoV counterpart. CoV S protein can bind distinct host factors, serving as receptor or co-receptor, to mediate viral entry. It has been well characterized that angiotensin-converting enzyme 2 (ACE2), dipeptidyl peptidase 4 (DPP4, CD26), and APN are the cellular receptors for SARS-CoV/SARS-CoV-2/common cold human coronavirus NL63 (HCoV-NL63) [9,56], MERS-CoV [57], and TGEV/PRCV/HCoV-229E/feline coronavirus (FCoV)/canine coronavirus (CCoV)/feline infectious peritonitis virus (FIPV)/PDCoV [58], respectively. It is highly possible that one or more critical sites within PDCoV S protein RBD are responsible for the loss of enteric tropism, resulting in the altered tropism of chimeric icPDCoV-S_HKU17_ and icPDCoV-RBD_ISU_. Additionally, the use of APN as the cellular receptor for chimeric viral entry was changed upon S protein or RBD replacement. Our study further confirmed that PDCoV can use other receptors besides APN to infect cells [19,21,22,27]. APN was not needed for icPDCoV-S_HKU17_ replication, suggesting that the S proteins from SpDCoV HKU17 may utilize other cellular proteins as the receptor. Moreover, the viral replication cycle is complex requiring various host factors to enable efficacious infection. Enhanced icPDCoV-RBD_ISU_ infection upon APN-knockout in ST cells may also provide a clue for the hypothesis that DCoVs can use a broad range of receptors, corresponding to its broad host ranges and infection in both birds and multiple mammals [59].

Concerns have escalated about CoV cross-species spillovers, as the pandemic SARS-CoV-2 jumped from bats or other mammals to humans, and are continually transmitted from humans to other species, such as felids and mink [52,60,61]. PDCoV is hypothesized to originate from avian species, possibly sparrows, that frequently congregate on pig farms or contaminate pig feed sources with their droppings. Therefore, it is crucial to understand the role of SpDCoVs in interspecies transmission of DCoV, which also serves as a model for better understanding the molecular mechanisms of cross-species spillovers of CoVs. In summary, our study generated a reliable reverse genetics platform for a virulent PDCoV OH-FD22 strain. Utilizing this platform, two chimeric viruses icPDCoV-S_HKU17_ and icPDCoV-RBD_ISU_ were constructed. They demonstrated an attenuated phenotype in pigs with loss of tropism for the intestinal tract. It will be very interesting to next determine whether these avian/swine chimeric viruses can still infect poultry.

## Figures and Tables

**Table 1:** General litter information and clinical signs of piglets after inoculation.

Inoculation Group	No. of Pigs	Inoculation Route (No. of Pigs)	Diarrhea Rate (%) ^b^	Onset of Diarrhea (dpi) ^a,c^	Mean Cumulative Fecal Consistency Score of Each Pig ^b,c,d^	Duration of Diarrhea (Days) ^a^	Peak Nasal RNA Shedding Titer (log_10_ Copies/mL) [dpi] ^a,e^	Peak Fecal RNA Shedding Titer (log_10_ Copies/mL) [dpi] ^a,e^
icPDCoV	4	Oral (4)	100 A	1.50 ± 0.58	2.78 ± 2.12 A	10.5 ± 0.58	6.88 ± 0.70 [2.5]	8.375 ± 0.81 [1.5]
OH-FD22	3	Oral (3)	100 A	2.00 ± 0.00	1.88 ± 1.76 A	10 ± 0.00	6.85 ± 1.46 [2]	8.92 ± 0.25 [2]
icPDCoV-S_HKU17_	5	Oral (3) Oronasal (2)	0 B	ND	0.22 ± 0.19 B	ND	6.06 ± 1.42 [4]	ND
icPDCoV-RBD_ISU_	2	Oral (2)	0 B	ND	0.5 ± 0.00 B	ND	5.08 [2] ^f^	ND
Mock	2	Oral (2)	0 B	ND	0.5 ± 0.50 B	ND	ND	ND

^a^ ND, not detected. ^b^ Different letters indicate a significant difference between groups (*p* < 0.05). Data were determined at 1 to 11 dpi. ^c^ Fecal consistency was scored as follows: 0, solid, 1, pasty, 2, semiliquid; and 3, liquid. Score of ≥ 2 was considered diarrhea. ^d^ Mean value = (sum of fecal consistency scores for each piglet)/N, where N is the number of surviving piglets in the period. Scores were calculated for piglets at 1 to 5, 7, 9, and 11 dpi. ^e^ The detection limit of RT-qPCR is 4.8 log_10_ GE/mL. ^f^ Not enough data to calculate standard deviation.

## Data Availability

The data presented in this study are available in this article and on request from the corresponding author.
